# Anthropogenic Effects on Hydrogen and Oxygen Isotopes of River Water in Cities

**DOI:** 10.3390/ijerph16224429

**Published:** 2019-11-12

**Authors:** Xiangnan Li, Baisha Weng, Denghua Yan, Tianling Qin, Kun Wang, Wuxia Bi, Zhilei Yu, Batsuren Dorjsuren

**Affiliations:** 1 State Key Laboratory of Simulation and Regulation of Water Cycle in River Basin, China Institute of Water Resources and Hydropower Research, Beijing 100038, China; lixn0555@163.com (X.L.);; 2 Water Resources Department, China Institute of Water Resources and Hydropower Research, Beijing 100038, China; 3 College of Hydrology and Water Resources, Hohai University, Nanjing 210098, China; 4 Institute of Water Resources and Hydrology Department of Hydraulic Engineering, Tsinghua University, Beijing 100084, China; 5 Department of Environment and Forest Engineering, School of Engineering and Applied Sciences, National University of Mongolia, Ulaanbaatar 210646, Mongolia

**Keywords:** stable isotopes, anthropogenic effect, city

## Abstract

Stable hydrogen and oxygen isotopes are important indicators for studying water cycles. The isotopes are not only affected by climate, but are also disturbed by human activities. Urban construction has changed the natural attributes and underlying surface characteristics of river basins, thus affecting the isotopic composition of river water. We collected urban river water isotope data from the Global Network for Isotopes in Rivers (GNIR) database and the literature, and collected river water samples from the Naqu basin and Huangshui River basin on the Tibetan Plateau to measure hydrogen and oxygen isotopes. Based on 13 pairs of urban area and non-urban area water samples from these data, the relationship between the isotopic values of river water and the artificial surface area of cities around rivers was analyzed. The results have shown that the hydrogen and oxygen isotope (δD and δ^18^O) values of river water in urban areas were significantly higher than those in non-urban areas. The isotopic variability of urban and non-urban water was positively correlated with the artificial surface area around the rivers. In addition, based on the analysis of isotope data from 21 rivers, we found that the cumulative effects of cities on hydrogen and oxygen isotopes have led to differences in surface water line equations for cities with different levels of development. The combined effects of climate and human factors were the important reasons for the variation of isotope characteristics in river water in cities. Stable isotopes can not only be used to study the effects of climate on water cycles, but also serve as an important indicator for studying the degree of river development and utilization.

## 1. Introduction

The stable hydrogen and oxygen isotope ratios of water molecules (δD and δ^18^O, respectively) are well-established, powerful, integrative recorders of key catchment processes and catchment water balance, as well as tracers of river recharge sources [1,2,3,4,5]. There are differences in the H–O isotopes values of different water sources under natural conditions. H–O isotopes can respond sensitively to environmental changes, reflecting isotope fractionation in the process of water phase transformation, which is widely used as an ideal indicator in water cycle research [6,7,8,9,10].

It has been well documented that the distribution of precipitation isotopes depends on the local temperature, latitude, elevation, and other climate or geographical factors, which can summarized as the following effects: (1) the elevation effect, (2) the rainfall effect, (3) the continental effect, (4) the temperature effect, (5) the latitude effect, and (6) the seasonal effect [1,2,11,12,13,14,15]. For instance, water at high elevation is highly depleted of heavy isotopes because of Rayleigh distillation and orography [12,16]. Regarding the continental effect, the isotopic composition of rainfall tends to be gradually depleted of heavier isotopes away from coastlines. Regarding the temperature effect, isotopic variations in precipitation have been correlated with mean surface air temperatures [11,15]. In addition, heavy isotopes always decrease with increasing latitude [11]. There is also seasonal variation in isotopic composition globally [2]. The identification of these climatic effects on H–O isotopes is important for better understanding of hydrological cycles and climate change.

However, these effects only consider climate factors and ignore anthropogenic effects. Anthropogenic effects greatly change the natural attributes of rivers, which may affect the isotopic composition in river water [17,18]. In addition, stable isotope analyses provide important information to water managers within large cities and the spatial and vertical understanding of the isotopes in water supplying urban systems can be used as a management tool to provide information on the origins and evaporative history of urban water supplies [19,20]. In urban areas, stable isotope research is also increasingly concerned with the impact of human activities [21,22].

In recent decades, with the increasing demand for water in society, a large number of reservoir projects have been built on rivers, which have caused about 70% of the world’s rivers to be intercepted by dams [17]. After damming, the dynamic conditions, retention time, and the mixing mode of the water source undergo significant changes [23,24,25]. Reservoir projects change river flow into the sea, a river’s temporal and spatial characteristics, the natural environment, and a river’s biogeochemical processes [26,27]. Recently, some scholars have investigated the impact of reservoir projects or cascade dams on H–O isotopes [28,29,30,31].

In addition, urbanization has changed the conditions of the underlying surface of river basins, resulting in changes in the temporal and spatial distribution of infiltration, surface runoff, and evaporation [32,33,34]. Water withdrawal, reuse of reclaimed water, and sewage discharge have also changed the nutrients, biochemical characteristics, and aquatic ecosystem function. A survey of global rivers revealed a state of moderate to high threat, with little evidence for turnaround with an ever-increasing population and rising water demands [35]. However, there is less concern about the local or cumulative effects of different cities around rivers on H–O isotopes, and no clear influencing factors have been identified for these effects. With the expansion of cities, how human activities will affect the H–O isotopes of rivers is a problem worth exploring.

To monitor the spatial–temporal variation in δD and δ^18^O values of river water, the International Atomic Energy Agency (IAEA) has established a global network for isotopes in rivers (GNIR) [4]. Based on this database, data from literature, and actual sampling points, this study mainly answers the following key questions: (1) What is the local effect of cities on hydrogen and oxygen isotopes? (2) What is the cumulative effect of cities on the hydrogen and oxygen isotopes of river water? (3) What are the factors related to these effects?

## 2. Data and Methods

### 2.1. Data

The main sources of δD and δ^18^O data in this paper are the GNIR database, literature data, and the actual sampling points for the Tibetan Plateau. In order to supplement the river water isotope samples of the GNIR database at high altitudes, the study collected 30 water samples in the Chenqu River in Tibet, China, and the Huangshui River in Qinghai Province in May and November 2018, respectively. The river water samples were mainly taken from the middle of the river or flowing water to ensure that the river water was well mixed. Immediately after the water sample was collected, it was placed in a 10 mL high-density polyethylene bottle, and the bottle cap was tightened, sealed with a sealing film, and then placed in a refrigerator to prevent evaporation of the isotopes. In the Agricultural Environment Stable Isotope Laboratory of the Chinese Academy of Agricultural Sciences, an L2130-i ultra-precision liquid water and water vapor isotope analyzer (Picarro) was used for analysis and testing. The stable isotope ratio is expressed in terms of the thousandth difference relative to the Vienna Standard Mean Ocean Water standard (V-SMOW). The analytical precision is better than ± 0.025‰ for δ^18^O and ± 0.1‰ for δD. The sample measurement method was a standard high precision method: 6 probes per sample, and the average of 3 probes was taken as the test result.

The entire data set includes 1928 river water samples from the GNIR database, 72 river water samples from the literature, and 30 water samples from actual sampling points. The information for these data is explained in detail in the Supplementary Tables, including isotope values, geographical coordinates, acquisition time, river name, and the data source. For the data from GNIR, because the water samples were analyzed at so many different laboratories and different analytical methods were used over many years, the analytical error can be assumed to be in the order of ± 0.2‰ for δ^18^O and ± 2‰ for δD [4]. For the data in the literature, we also included the analytical precision of the data in Table 1.

**Figure 1:**
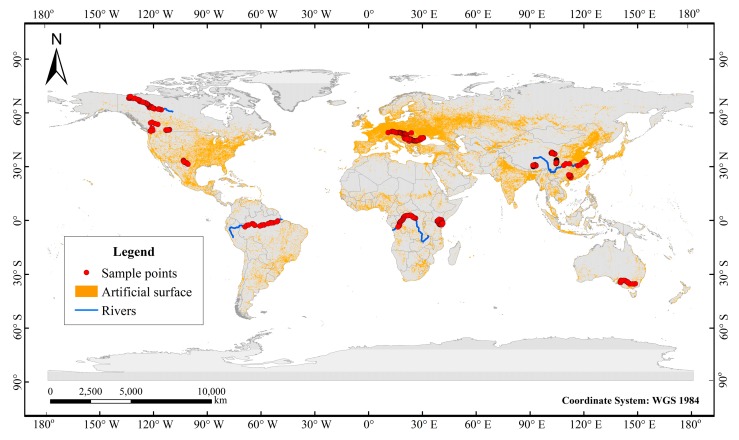
The locations of the sample points and rivers corresponding to Table 2.

In order to study the local effect of a city, we selected 13 pairs of urban area and non-urban area water samples from the above data set (Table 1). These samples were carefully selected. Each pair of samples is spatially continuous, with no inflow of other tributaries between them to reduce interference from other factors. In addition, in order to study the cumulative effect of cities on isotopes, 311 samples from 21 rivers around the world were selected (Table 2). Since most urban areas are concentrated in the mid-latitudes, in order to analyze the impact of cities on river isotopes at the same latitudes, we also indicate the latitude range of each river. Figure 1 shows the locations of the sample points and rivers corresponding to Table 2. The samples from each river were collected during the same period. All the stable isotope values are expressed in terms of the thousandth difference relative to the Vienna Standard Mean Ocean Water standard (V-SMOW).

The land use data in this paper is derived from the land use raster data of globe30, which is produced by the Chinese government using remote sensing data (www.globeland30.com). Ten types of land use were included in the data: land surface waters, wetlands, woodlands, grasslands, shrubs, artificial surfaces, arable land, glaciers and permanent snow, tundra, and bare land. Among them, artificial surface areas are the main areas containing urban construction.

### 2.2. Methods

In this study, we selected the area of the city (artificial surface area) as an indicator of the city size around the river. Artificial surface area is the key factor affecting the climate characteristics of urban areas. Especially in cities with more artificial surface area, the urban surface atmospheric temperature is often higher than the surrounding non-urban areas [39].

**Figure 2:**
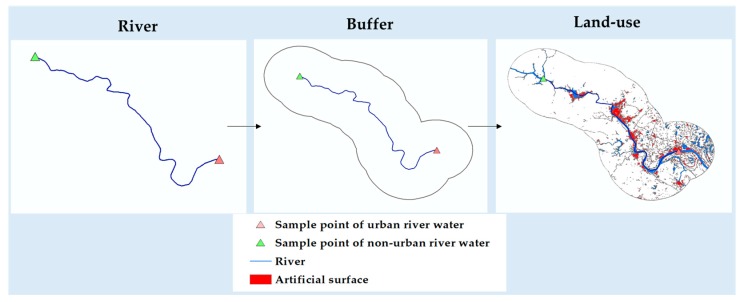
The method used to calculate the artificial surface area around a river.

Based on the 13 pairs of samples from cities, we intercepted the river sections between the sampling points of urban area and non-urban area water, made 10-km, 20-km, and 50-km buffer zones for the river sections, and then calculated the artificial surface area of the buffer zones. The specific method is shown in Figure 2. The indicators of the cities in Table 1 are shown in Table 3.

In order to analyze the cumulative effects of the cities on the δD and δ^18^O of the river water samples, we divided 21 rivers into three levels according to a similar method as above. We intercepted the river sections between the most upstream and downstream sampling points of 21 rivers, and made a 20-km buffer zone for each river section, and then calculated the proportion of the area of the artificial surface in the buffer out of the entire buffer area. It can be considered that a river with a greater proportion has a higher degree of development and utilization (i.e., more cities around the river). According to this classification system, 21 rivers were divided into three grades: rivers with a proportion of less than 1% are level 1, with most of these being near-natural rivers, which are less disturbed by humans; rivers with a proportion greater than 1% and less than 5% are level 2, and such rivers are subject to weak human disturbances; rivers with a ratio of more than 5% are level 3, and most of these rivers pass through large- or medium-sized cities, which are strongly disturbed by human activities. The classification and characteristics of the rivers are shown in Table 2.

The artificial surface area in the buffer zone was selected as the key indicator to study the difference in water isotope values between urban and non-urban areas. Based on Statistical Product and Service Solutions (SPSS) software, the linear fitting equation model was used to analyze the relationship between the variation of δD (δ^18^O) and the key indicators. Considering that the ordinary least squares regression does not take into account the analytical error on the X-variable, the error-in-variable regression model was used [40]. The error-in-variable regression model was based on Origin Pro software. For the linear regression test, we completed the analysis based on the variance analysis table. There are several methods used to test the significant of liner regressions. These methods are not only limited to analysis of variance (ANOVA), but also include analysis of relationship between the Pearson’s correlation coefficient (r) and sample dimension (N) and lack-of-fit test. Based on the SPSS model, we performed variance analysis tests for all linear regressions. Furthermore, the Student’s t-test was used to check the difference between the two regression slopes (intercepts) [41].

## 3. Results

### 3.1. Local Effects of the Cities

**Figure 3:**
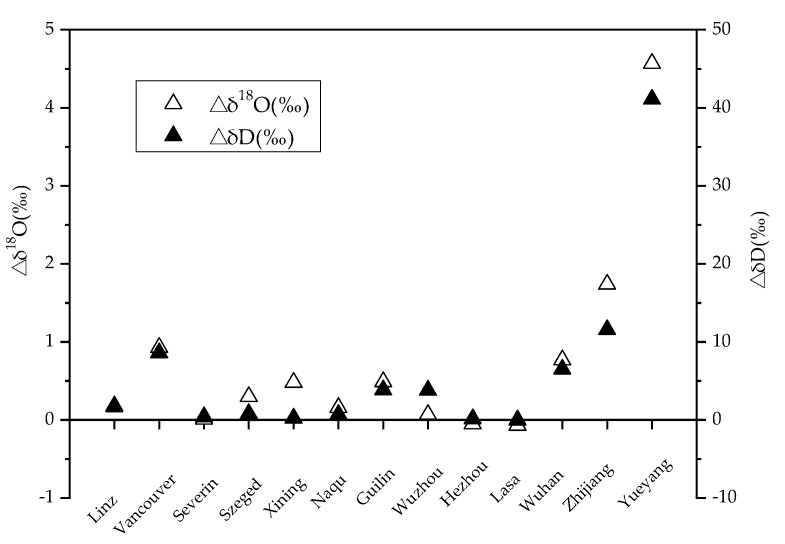
The △δD and △δ^18^O values from the cities. Here, △δD (or △δ^18^O)represents the difference between the δD (or δ^18^O) value of urban river water and the δD (or δ^18^O) value of non-urban river water.

Since the urban water and the non-urban water samples were basically in the same geographical positions, calculating the difference between them as a criterion could also effectively eliminate the impact of different climates or different sources. According to the data in Table 1, the isotopic values of urban water isotopes were generally higher than those of non-urban water isotopes (see Figure 3).

In order to analyze the deviation of δD and δ^18^O values of urban and non-urban river water samples (△δD and △δ^18^O, respectively), the linear fitting equation model was used to analyze the relationship between the deviation and the artificial surface area, and significance analysis was carried out.

**Figure 4:**
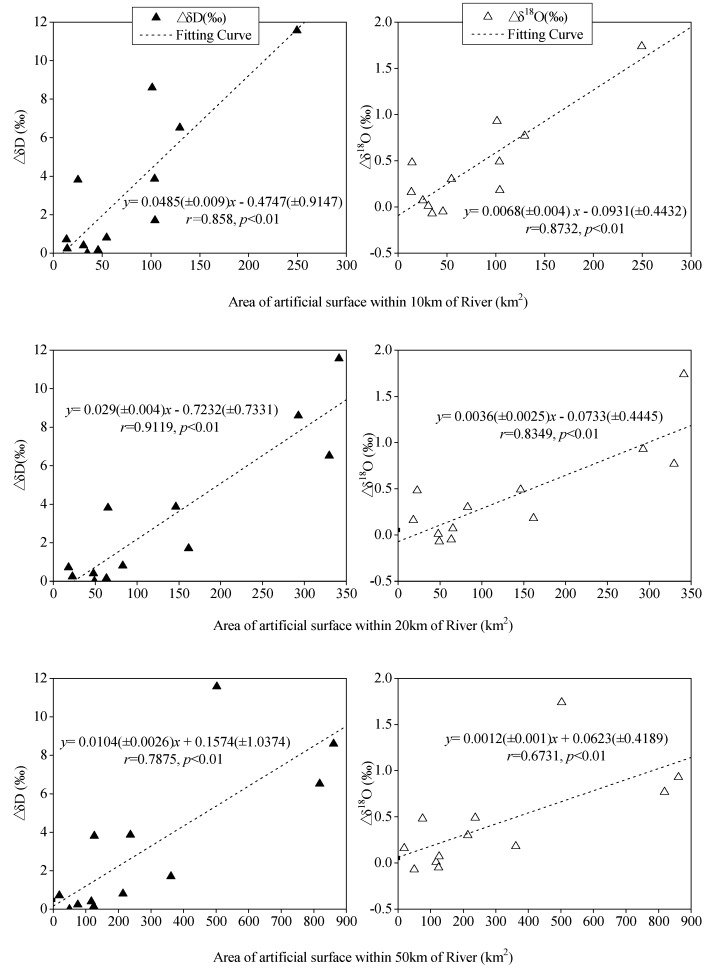
Relationship between △δD or △δ^18^O value and artificial surface areas of the river in the range of 10 km, 20 km, and 50 km.

As shown in Figure 4, it can be seen that except for Yueyang City, the △δD or △δ^18^O of urban river water and non-urban samples had a significant positive correlation with the artificial surface area around the river, and the closer the artificial surface area was to the river, the stronger the impact on the river. The effect of artificial surface area on the δD and δ^18^O was slightly different. As the south of Yueyang City is adjacent to the Dongting Lake, the interaction between lake and city led to a higher δD and δ^18^O values at the sampling point in Yueyang City, which deviated significantly from the fitting curve. Therefore, this point was treated as an abnormal point in the analysis, and was not used as a sample point of the linear fitting model.

These results indicate that the expansion of a city not only significantly changes the climate around the city, but also affects the evaporation fractionation of stable isotopes in urban rivers. The higher density of the construction land is, the stronger the stable isotopic evaporation fractionation. Therefore, for urban rivers, δD and δ^18^O can be used as important indicators to reflect the impact of cities around a river on its water cycle.

### 3.2. Cumulative Effects of the Cities

Based on the data of rivers of different development levels (Table 2), the cumulative effect of the city was analyzed.

Craig originally established a linear relationship between δD and δ^18^O in atmospheric precipitation, namely the meteoric water line (MWL), which is of great significance for studying stable isotope changes in the water cycle [42]. Rozanski et al. analyzed the global meteoric water line (GMWL) from 206 samples from around the world through the global International Atomic Energy Agency (IAEA) network station [11]. Gourcy et al. used more recent data (1961-2000) to establish the GMWL, described by the following equation [43]:

δD = [(8.14 ± 0.02) δ^18^O] + [10.9 ± 0.2] (1)

The difference between the MWL of different regions and GMWL is mainly affected by the different water vapor sources, evaporation of raindrops, and local water vapor recirculation.

**Figure 5:**
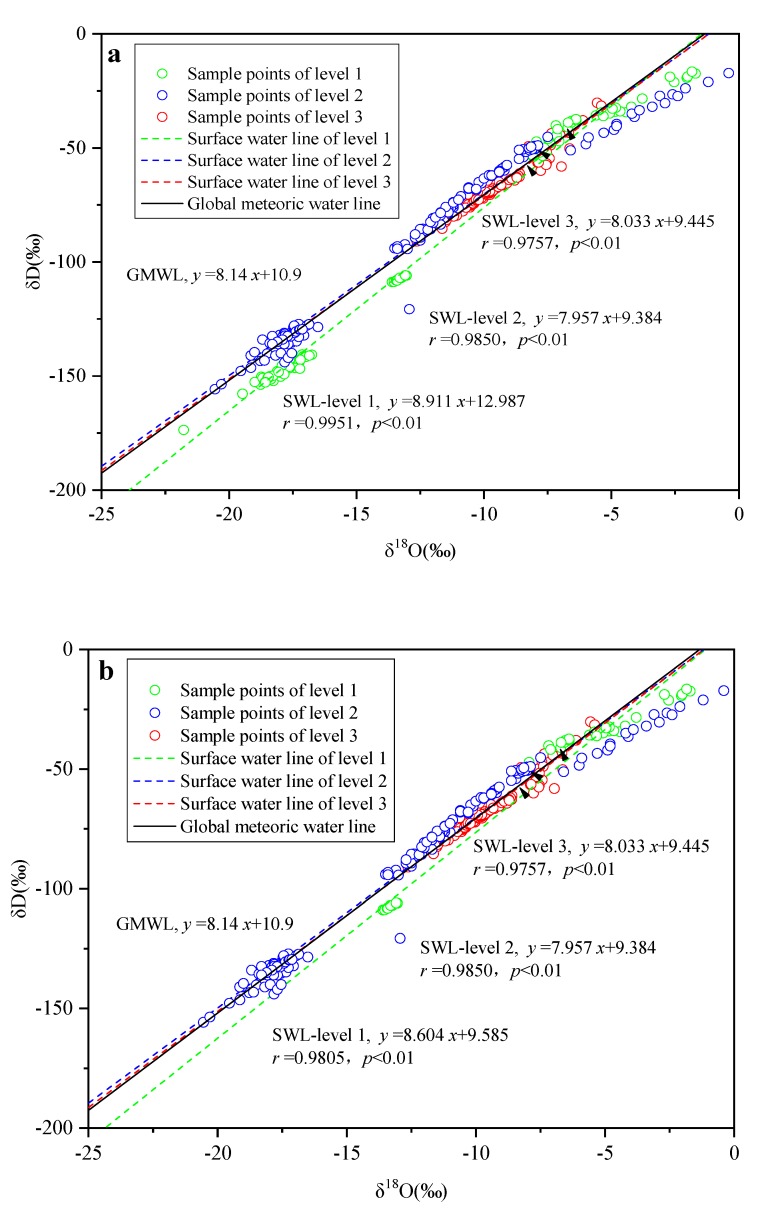
Relationship between δD and δ^18^O of river water of different levels: (**a**) all rivers; (**b**) mid-latitude rivers. Note: SWL = surface water line; GMWL = global meteoric water line.

The surface water line equations of rivers with different human activity interference levels were calculated, and in order to eliminate the latitude effects of isotopes in rainwater, the surface line equations of three levels of rivers at mid-latitude (30° N–60° N or 30° S–60° S) were also obtained, as shown in Figure 5.

For all rivers, the surface waterline equations for the rivers are:

Level-1: δD = ((8.911 ± 0.156) δ^18^O) + (12.987 ± 2.105) (2)

Level-2: δD = ((7.957 ± 0.134) δ^18^O) + (9.384 ± 1.786) (3)

Level-3: δD = ((8.033 ± 0.529) δ^18^O) + (9.445 ± 5.214) (4)

For mid-latitude rivers, the surface waterline equations for the rivers are:

Level-1: δD = ((8.604 ± 0.359) δ^18^O) + (9.585 ± 2.934) (5)

Level-2: δD = ((7.957 ± 0.134) δ^18^O) + (9.384 ± 1.786) (6)

Level-3: δD = ((8.033 ± 0.529) δ^18^O) + (9.445 ± 5.214) (7)

It can be found that the higher the level of the rivers, the slope and intercept of the surface water line (SWL) are smaller. Based on Student’s t-test method, we verified the significance of the difference in slope and intercept of different surface waterline equations. The results showed that there were significant differences in slopes and intercepts between natural rivers (Level 1) and rivers that were heavily disturbed by human activities (Level 2, Level 3), whether for all rivers or mid-latitude rivers only. The difference between level 2 and level 3 rivers was not significant.

These results reflected the stronger evaporation and fractionation of H–O isotopes in river water as the urban construction disturbances increase. The effect was clearly found for both global and mid-latitude rivers; that is, urban construction may enrich the heavy H–O isotopes in rivers. Similar to the effect of reservoirs, this effect can be accumulated by a succession of cities [31]. With the development of urban construction, the SWL equation may gradually change to the SWL level 3 equation.

Although cities promoted the evaporation fractionation of H–O isotopes in river water, this effect was gradually weakened by the influence of rainfall recharge and river water mixing. Therefore, the δD and δ^18^O values showed a jagged trend from upstream to downstream sections of a river [31].

## 4. Discussion

### 4.1. Analysis of the Impact Factors

The research in this paper shows that the anthropogenic effects changed the isotopic composition of river water. This study found that in urban areas, the hydrogen and oxygen isotopic values were significantly higher than in non-urban area, and the △δD or △δ^18^O values had a strong positive correlation with the artificial surface area. Hydrogen and oxygen isotopes are key tracers for studying the water cycle, and understanding the factors affecting the value of isotopes is crucial for future research.

**Figure 6:**
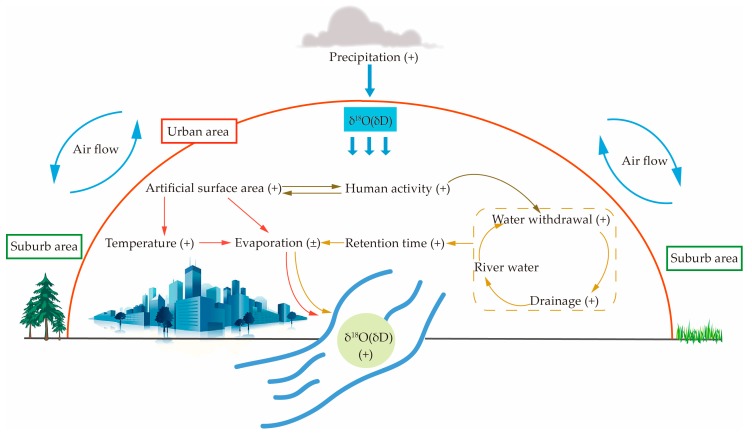
Schematic diagram of anthropogenic effects on δD and δ^18^O values of river water in cities.

Figure 6 shows the schematic diagram of anthropogenic effects on δD and δ^18^O values of the river water in cities. There are two main reasons for this impact: climate factors and human activity factors.

For climate factors, the increase in the artificial surface area of a city enhances the heat island effect. The urban heat island effect can affect meteorological elements such as temperature and rainfall in urban areas. In cities with high-speed construction, the urban surface atmospheric temperature is often higher than the surrounding non-urban areas. The heat islands effect impacts the formation and movement of clouds, which leads to changes in rainfall mechanisms in local areas [44,45]. It is generally believed that urban heat island effects can increase urban rainfall [46]. The increase in local temperature may also promote the isotope evaporation fractionation of river water. However, there is some evidence that heavily urbanized areas have altered evapotranspiration regimes due to the removal of vegetation and the decrease of water retention and soil storage. A study by Albrecht (1974) that assessed the hydrology of an urban catchment found that when the urbanized surface grew, a 50% increase in total runoff was observed, as well as a reduction by a factor of two for both percolation to groundwater and actual evapotranspiration rates [47]. Therefore, the impact of urban environmental factors on river water isotopes is quite complicated.

For human activity factors, as the impact of human activities on the water cycle gradually increase, the water cycle clearly exhibits the dualistic characteristics of nature–society, while in urban areas, the social water cycle dominates [48,49]. In urban areas, river water experiences many processes, such as water withdrawal, water use, and drainage, and the promotion of water-saving measures leads to the recycling of water. These processes make the retention time of the water in an urban watershed longer than that of a natural river basin. These effects may lead to higher values of hydrogen and oxygen isotopes in urban river waters. However, the role of human activities in cities is complex. Not only the size of the city, but also factors such as the population of the city, the source of the water, and the flow rate of the river affect the value of the river water isotopes.

Given the precision of existing data and the complexity of the role of cities, the correlation of these factors is difficult to explain clearly. More in-depth research is needed in the future. In summary, a variety of complex factors cause the value of H–O isotopes in rivers in cities to be larger than those in non-urban areas.

### 4.2. Similarities and Differences between City Effect and Reservoir Effect

This effect of cities on H–O isotopes is similar to the effect of a reservoir. After a reservoir is constructed, the surface area and retention time of the water increase, which promotes the evaporation fractionation of H–O isotopes, resulting in a large difference in H–O isotope characteristics between reservoir water and inflowing water. In rivers with more dams, the overall retention time of the rivers will increase, resulting in the “age” of the entire river being older than it was previously [50]. For example, some studies have shown that the current river “age” in the Yangtze River Basin in China is about 1.4 months older than it was in the 1980s [30]. Similar to our research, the “age” of the rivers flowing through a city will be older than in natural rivers. This is because the observed enrichment of heavy isotopes in urban water actually reflects the fact that these waters are subjected to stronger or longer evaporation fractionation.

Moreover, the values of H–O isotopes in the rivers with more reservoirs will also show a jagged increase along the river from upstream to downstream sections. Studies have also shown that the effect of reservoirs has a strong correlation with the retention time of the river water [30,31]. This may be similar to the role of human factors in cities.

### 4.3. Other Factors to be Studied

In fact, the anthropogenic effects on H–O isotopes from cities are more complicated. This paper only considers the evaporation changes caused by the heat island effect of a city and the changes in river water retention time caused by water withdrawal. In fact, factors including the population of the city, the source of river water, and the natural properties of the river, such as flow rate, runoff, and the width of the riverbed, can also affect the evaporation of the river, thereby changing the composition of the isotopes. If a river has little runoff, a slow flow rate, and a wide river channel, this will lead to a large amount of evaporation of the river water [24,30]. The discharge of domestic sewage and industrial wastewater may also change the hydrogen and oxygen isotope characteristics of local river water to some extent. Furthermore, local climate change caused by urbanization may cause isotope changes in urban river water caused by many factors. Urbanization changes the temperature, rainfall and atmospheric pressure in cities, factors which are also closely related to H–O isotopes in precipitation. From a global perspective, the δ^18^O in mid–high latitude continental rainwater has a significantly positive correlation with temperature [51], while at the middle and low latitudes, there are different positive and negative correlation zones [51]. In the global water cycle, the rivers usually inherit the H–O isotope signatures of the precipitation. Therefore, the cities indirectly affect the characteristics of urban river water H–O isotopes by affecting the precipitation isotopes.

With city development, the H–O isotopes of river water may undergo irreversible changes. Since the effect of cities on the H–O isotopes of river water is quite complicated, this study does not conduct an in-depth analysis of the source and climatic conditions of stable river stable isotopes, and it is necessary to continue research in the future.

## 5. Conclusions

As important indicators, hydrogen and oxygen isotopes have been used in previous studies to analyze the basic characteristics of water and the sources of water supply. Moreover, the research on the influencing factors of hydrogen and oxygen isotopes is mostly based on climatic factors, such as latitude, temperature, and source, and less consideration is given to the influence of human activities on the hydrogen and oxygen isotopes of river water. As human activities intensify, the anthropogenic effects cannot be ignored when studying the water cycle.

Based on the GNIR database, literature data, and actual sampling data, this study analyzed the changes in the values of hydrogen and oxygen isotopes in urban river water. The main conclusions are as follows.

1. The values of the hydrogen and oxygen isotopes of urban river are generally higher than that of the corresponding non-urban river water.
2. The isotopic variability value of urban and non-urban water is positively correlated with the artificial surface area around the river.
3. The more artificial surface area around the river, the smaller the slope and intercept of the surface water line equation.

Although the impact of human activities will be gradually weakened because of the influence of rainfall or river convergence, with the intensification of urban construction, the hydrogen and oxygen isotopes of river water may undergo greater changes. It is of great importance to study the anthropogenic effects on hydrogen and oxygen isotopes in the future.

## Figures and Tables

**Table 1:** Stable hydrogen and oxygen isotope ratios (δD and δ^18^O, respectively) of urban and non-urban river water samples in cities. Note: GNIR = global network for isotopes in rivers.

City	Water in Urban Area				Water in Non−Urban Area				Analytical Precision		Sample Date	Data Source
	δ^18^O (‰)	δD (‰)	Latitude (°)	Longitude (°)	δ^18^O (‰)	δD (‰)	Latitude (°)	Longitude (°)	δ^18^O (‰)	δD (‰)		
Linz	−10.65	−76.1	48.255	14.417	−10.83	−77.8	48.524	13.693	±0.2	±2	2007-08-18	GNIR
Vancouver	−16.45	−125.1	49.214	−122.782	−17.38	−133.7	49.180	−122.567	±0.2	±2	2009-07-28	GNIR
Drobeta-Turnu Severin	−9.86	−69.9	44.599	22.714	−9.87	−70.3	44.692	22.407	±0.2	±2	2007-09-11	GNIR
Szeged	−8.61	−62.7	46.255	20.202	−8.91	−63.5	46.129	20.099	±0.2	±2	2007-08-31	GNIR
Xining	−7.991	−50.375	36.632	101.783	−8.472	−50.609	36.570	101.874	±0.025	±0.1	2018-11-9	Sampling points
Naqu	−13.44	−108.102	31.479	92.042	−13.6	−108.809	31.528	92.037	±0.025	±0.1	2018-05-20	Sampling points
Guilin	−5.04	−29.98	25.281	110.301	−5.53	−33.84	24.779	110.495	±0.2	±0.6	2015-01-15	Xu et al., 2017 [36]
Wuzhou	−6.16	−42.01	23.468	111.31	−6.23	−45.82	23.417	111.493	±0.2	±0.6	2015-01-17	Xu et al., 2017 [36]
Hezhou	−5.19	−33.93	24.409	111.505	−5.14	−34.07	23.964	111.735	±0.2	±0.6	2015-01-17	Xu et al., 2017 [36]
Lasa	−17.32	−129	29.642	91.113	−17.25	−129	29.667	91.301	±0.1	±1	2009-08	Yu et al., 2010 [37]
Wuhan	−6.64	−38.58	30.568	114.294	−7.41	−45.1	30.437	114.189	±0.1	±0.8	2003-01	Sun, 2007 [38]
Zhijiang	−9.9	−71.57	30.416	111.766	−11.64	−83.14	30.962	110.755	±0.1	±0.8	2003-01	Sun, 2007 [38]
Yueyang	−5.55	−29.76	29.452	113.143	−10.12	−70.86	29.786	112.861	±0.1	±0.8	2003-01	Sun, 2007 [38]

**Table 2:** The classification and characteristics of the rivers.

Level	Code	River	Length of the Reach (km)	Artificial Surface Area (km^2^)	Proportion of the Artificial Surface Area	Latitude
1	1	Athi	327.08	36.66	0.3%	0°–30° S
	2	Amazon	1176.98	99.96	0.2%	0°–30° S
	3	Galana	174.1	3	0.05%	0°–30° S
	4	Mackenzie	1719.91	66.73	0.1%	60° N–90° N
	5	Murray	1908.21	247.2	0.5%	30° S–60° S
	6	Solimoes	1641.26	70.99	0.1%	0°–30° S
	7	Tana	868.69	30.02	0.1%	0°–30° S
	8	Chenqu	18.98	12.01	0.6%	30° N–60° N
2	9	Congo	1720.26	672.17	1%	0°–30° S
	10	Fraser	1359.87	1208.21	2.8%	30° N–60° N
	11	Min	329.92	156.01	1.3%	30° N–60° N
	12	Oldman	290.19	128.79	1.4%	30° N–60° N
	13	Pecos	556.61	471.98	2.6%	30° N–60° N
	14	Huangshui	212.48	190.66	2%	30° N–60° N
	15	Beichuan	114.75	133.25	2.5%	30° N–60° N
	16	Lhasa River	255.63	97	1%	30° N–60° N
3	17	Danube	3034.89	6573.2	6.6%	30° N–60° N
	18	Great Morava	180.81	377.86	6.1%	30° N–60° N
	19	Sava	225.02	490.59	7.5%	30° N–60° N
	20	Tisza	838.37	1435.87	5.7%	30° N–60° N
	21	Yangtze	2233.13	3865.3	5.2%	30° N–60° N

**Table 3:** The basic characteristics and indicators of the cities.

City	River Basin	Length of River Reach (km)	Artificial Surface Area (km^2^)		
			**10 km**	**20 km**	**50 km**
Linz	Danube	88.84	104.1	161.65	361.54
Vancouver	Fraser	18.60	101.26	292.78	860.98
Drobeta-Turnu Severin	Danube	32.52	30.98	48.02	116.73
Szeged	Tisza	23.97	54.62	83.12	213.94
Xining	Huangshui	12.56	14.21	22.80	75.25
Naqu	Chenqu	13.49	13.49	18.32	18.51
Guilin	Lijiang	81.71	103.68	146.38	237.11
Wuzhou	Xijiang	22.83	25.19	65.43	125.79
Hezhou	Hejiang	88.79	45.95	63.47	124.34
Lhasa	Lhasa River	22.09	34.81	49.27	49.65
Wuhan	Yangtze	19.20	129.49	329.57	818.33
Zhijiang	Yangtze	172.30	249.52	341.25	502.43
Yueyang	Yangtze	86.67	46.9	121.28	252.17

## Supplementary Materials

The following are available online at https://www.mdpi.com/1660-4601/16/22/4429/s1, S1: isotope data.
